# Protective and therapeutic potency of N-acetyl-cysteine on propionic acid-induced biochemical autistic features in rats

**DOI:** 10.1186/1742-2094-10-42

**Published:** 2013-03-27

**Authors:** Abeer M Aldbass, Ramesa Shafi Bhat, Afaf El-Ansary

**Affiliations:** 1 Department of Biochemistry, College of Science, King Saud University, P.O Box 22452, Riyadh, 11495, Saudi Arabia

**Keywords:** Comet DNA, Glutathione-s transferase, Interferon-γ, N-acetyl-cysteine, Propionic acid, Serotonin

## Abstract

**Background:**

The investigation of the environmental contribution for developmental neurotoxicity is very critical. Many environmental chemical exposures are now thought to contribute to the development of neurological disorders, especially in children. Results from animal studies may guide investigations of human populations towards identifying either environmental toxicants that cause or drugs that protect from neurotoxicity and may help in treatment of neurodevelopmental disorders.

**Objective:**

To study both the protective and therapeutic effects of N-acetyl cysteine on brain intoxication induced by propionic acid (PPA) in rats.

**Methods:**

Twenty-eight young male Western Albino rats were enrolled in the present study. They were grouped into four equal groups, each of 7 animals. Group 1: control group, orally received only phosphate buffered saline; Group 2: PPA-treated group, received a neurotoxic dose of of PPA of 250 mg/kg body weight/day for 3 days; Group 3: protective group, received a dose of 50 mg/kg body weight/day N-acetyl-cysteine for one week followed by a similar dose of PPA for 3 days; and Group 4: therapeutic group, treated with the same dose of N-acetyl cysteine after being treated with the toxic dose of PPA. Serotonin, interferon gamma (IFN-γ), and glutathione-s-transferase activity, together with Comet DNA were assayed in the brain tissue of rats in all different groups.

**Results:**

The obtained data showed that PPA caused multiple signs of brain toxicity as measured by depletion of serotonin (5HT), increase in IFN-γ and inhibition of glutathione-s-transferase activity as three biomarkers of brain dysfunction. Additionally Comet DNA assay showed remarkably higher tail length, tail DNA % damage and tail moment. N-acetyl-cysteine was effective in counteracting the neurotoxic effects of PPA.

**Conclusions:**

The low dose and the short duration of N-acetyl-cysteine treatment tested in the present study showed much more protective rather than therapeutic effects on PPA-induced neurotoxicity in rats, as there was a remarkable amelioration in the impaired biochemical parameters representing neurochemical, inflammatory, detoxification and DNA damage processes.

## Introduction

The impact of environmental chemicals on children's neurodevelopment is sometimes treated as an unimportant issue because of the insignificant clinical impairments. Such a judgment reflects a failure to distinguish between individual and population risk. The population impact of a risk factor depends on both its size of effect and distribution (or incidence/prevalence) [1]. The particular vulnerability of the developing nervous system for low-level exposure to chemicals is well established. It has been argued that a large number of environmental chemicals cause some degree of developmental neurotoxicity. However, for only a few of these, human evidence was able to suggest that exposure to chemicals could be contributing to neurodevelopmental disorders like autism, attention-deficit disorder, mental retardation or cerebral palsy [2].

Propionic acid (PPA) is an intermediate in cellular fatty acid metabolism. It is found in high levels in the gut, along with a number of other short chain fatty acids, such as acetate and butyrate, each of which is a product of enteric bacteria [3]. PPA may play a role in the behavioural, neuropathological and biochemical abnormalities observed in autism. It is capable of gaining access to the brain and inducing different adverse effects on the central nervous system [4]. Recent work reported the potency of PPA in altering dopamine, serotonin, and glutamate as neurotransmitter systems involved in the aetiology of neurodevelopmental disorders [5-7], partly via stimulating intracellular calcium release [8,9]. Additionally, alterations in the serotonin and dopamine systems have been implicated in abnormal social and motor behaviours [5,6,10], similar to the symptoms observed in both autistic patients and PPA animal models of MacFabe *et al.*[11]. PPA is also known to potentiate glutamatergic transmission [12], inhibit GABAergic transmission and potentiate the production of enkephalin [8,13] supportive of a potential mechanism for the enhanced excitation/reduced inhibition theory of autism [14]. Moreover, the inflammatory action of PPA was reported through the induction of interleukin-6 (IL-6), tumour necrosis factor-α (TNF-α) and interferon-γ (IFN-γ) as proinflammatory cytokines [7,11]. Recently, depletion of glutathione (GSH), lower activity of glutathione peroxidase and elevation of lipid peroxides are among the most persistent neurotoxic effects of orally administered PPA [3]. GSH has a critical role in both antioxidant defence and detoxification of xenobiotics for a broad range of environmental chemicals [15,16], including those implicated in autism spectrum disorders. Impairments in GSH-associated pathways suggest reduced cellular defence and are therefore considered as markers of increased oxidative stress [15,17].

Oxygen is both essential and toxic to all forms of aerobic life and the chemical versatility and reactivity of thiols play a key role in both aspects. Cysteine thiol groups have key catalytic functions in enzymes and are readily damaged by reactive oxygen species (ROS). Low-molecular-weight thiols provide protective buffers against the hazards of ROS toxicity. GSH is the small protective thiol in nearly all eukaryotes, but in prokaryotes the situation is far more complex [18]. Depletion of intracellular GSH appears to be critical for subsequent alterations in protein thiols and calcium homeostasis [19]. GSH depletion and the subsequent low stores of protein thiols result in both calcium release from intracellular stores and inhibition of calcium extrusion, producing a marked increase in cytosolic calcium concentration, which triggers cytotoxicity [19]. The brains of patients with autism spectrum disorders and animal models exhibited a reduction of GSH [10]. It was postulated to be of significance in the pathogenesis of this disease via oxidative damage [20,21]. Cysteine, the rate-limiting amino acid for GSH synthesis, was significantly decreased in autistics relative to the control children suggesting that GSH synthesis was insufficient to maintain redox homeostasis [16]. GSH depletion occurred without a change in GSSG (oxidised form of GSH), suggesting efflux of GSH out of the glia, perhaps with additional increase in the conversion of GSH to GSSG in response to the increased hydrogen peroxide formation [22]. At this point it is unclear whether free radical involvement in autism is a primary or secondary event in brain cell death, or whether or not it occurs early or late in the disease process.

Based on the fact that an impaired cysteine uptake from the plasma has been proposed secondary to a decrease in plasma GSH, this work was undertaken to investigate the protective and therapeutic effects of oral supplementation of N-acetyl-cysteine (NAC) to ameliorate the persistent biochemical autistic features induced in PPA-treated rats.

## Materials and methods

### Animals

This is an interventional experimental animal study performed on twenty-eight male western albino rats (45 to 60 g, approximately 21 days old). Rats were obtained from the Pharmacy College animal house at King Saud University. They were kept under standard conditions of temperature, 12-h dark/light cycle and were given free access to tap water and standard laboratory chow. After one week of acclimation, the rats were divided into four groups (seven rats in each group), namely the control group in which animals were fed normal diet during the experimental period; the PPA treated rats that received 250 mg/kg body weight/day for 3 days, in order to induce autistic features; the protective group which received 50 mg/kg/day of NAC for one week followed by oral PPA (250 mg/kg body weight/day for 3 days); and the therapeutic group that received NAC for one week after PPA oral dose for three days.

### Tissue preparation

At the end of the feeding trials, the rats were anesthetized with carbon dioxide and decapitated. The brain was removed from the skull and dissected into small pieces and homogenized in 10 times w/v bi-distilled water and kept at −80°C until further use for different biochemical analyses. A small piece of brain was kept separately for Comet DNA assay.

### Ethics approval and consent

This work was approved by the Ethical committee of Science College at King Saud University (Approval no 8/25/220358).

### Assay of serotonin

Serotonin was measured using an ELISA kit from Immuno-Biological Laboratories (IBL, Hamburg, Germany) [23]. Brain homogenate preparation (derivatization of serotonin to N-acyl-serotonin) was part of the sample dilution which was achieved by the incubation of the respective sample with the acylating reagent. The assay procedure followed the competitive ELISA protocols whereby competition takes place between biotinylated and non-biotinylated antigens for a fixed number of antibody binding sites. The amount of biotinylated antigens bound to the antibodies was inversely proportional to the N-acyl-serotonin concentration of the sample.

### Assay of IFN-γ

IFN-γ was measured using an ELISA kit, a product of Thermo Scientific (Rockford, IL, USA) [24] according to the manufacturer’s instructions. This assay employs a quantitative sandwich enzyme immunoassay technique that measures IFN-γ in less than five hours. The minimum level of rat IFN-γ detected by this product was less than 2 pg/mL.

### Determination of glutathione-S-transferase activity (GST)

GST activity was assessed using (Biovision, USA) assay kit, based upon the GST-catalysed reaction between GSH, GST substrate, and CDNB (1-chloro-2,4-dinitrobenzene). The GST-catalysed formation of GS-DNB produces a dinitrophenyl thioether which can be spectrophotometrically detected at 340 nm.

### Comet DNA assay

Single cell gel electrophoresis or Comet assay is one of the simple, sensitive and rapid methods for the detection and quantification of DNA damage [25]. Slides were prepared in duplicate per group and the test was performed for at least 3 different brain samples from each group. Cell suspension, about 4 × 10^6^ cells were mixed with 80 μL of 0.7% low-melting agarose in phosphate-buffered saline (PBS) at 37°C in a microtube, and then spread over a window microscopic slide. The slides were pre-coated with 150 μL of 0.5% normal-melting agarose in PBS, and were specially designed for this assay. Then, slides were placed immediately in cold lysis buffer, 2.5 M sodium chloride NaCl, 100 mM EDTA sodium salt Na_2_EDTA, 10 mM Tris (pH 10), and 1% Triton X-100, at 4°C for a minimal of 1 hr. After lysis, the slides were drained and placed in a horizontal gel electrophoresis tank surrounded by ice, and filled with fresh cold electrophoresis buffer (300 mM sodium hydroxide NaOH, 1 mM NaEDTA, pH 13). To allow DNA unwinding, the slides were kept in the high pH buffer for 20 min. After that, electrophoresis was carried out for 20 min at 25 V and 300 mA. The slides were then drained and flooded slowly with 3 changes of neutralization buffer (0.4 M Tris, pH 7.5) for 5 min each, and then stained with 30 mL of ethidium bromide (20 mg/L), and covered with cover slips. All those steps were performed under dimmed light in order to prevent additional DNA damage caused by visible light. A total of 50 randomly selected cells per slide were analysed. Imaging was done using a fluorescence microscope (Zeiss Axiovert L410 Inc., Jena, Germany), attached to a digital camera (Olympus Inc., Tokyo, Japan), and equipped with 549 nm excitation filter, 590 nm barrier filter, and a 100-W mercury lamp. The percentage of DNA in the comet tail "DNA damage" was automatically calculated [25].

Comets were randomly captured at a constant depth of the gel, avoiding the edges of the gel, occasional dead cells, and superimposed comets. DNA damage was measured as tail length (TL = distance of DNA migration from the centre of the body of the nuclear core), and tail intensity DNA (TI = % of genomic DNA that migrated during the electrophoresis from the nuclear core to the tail). By presenting all three parameters together, more information could be obtained on the extent of DNA damage.

### Statistical analysis

The data were analysed using the statistical package for the social sciences (SPSS, Chicago, IL, USA). The results were expressed as mean ± standard error of the mean (SEM). All statistical comparisons between the control and PPA-treated rat groups were performed using the one-way analysis of variance (ANOVA) test complemented with the Dunnett’s test for multiple comparisons. Significance was assigned at the level of *P* <0.05. Receiver operating characteristics (ROC) curve analysis was performed. Area under the curve (AUC), cut-off values, and degree of specificity and sensitivity were calculated. Pearson correlations were calculated.

## Results and discussion

Typically, an animal model is unlikely to replicate a human disease [10]. The utility of such models relates to the various types of validity that can be shown to exist for specific models. An emerging hypothesis in autism is the disequilibrium between oxidants and antioxidants in the body which leads to the accumulation of ROS. Originally ROS are removed by GSH, superoxide dismutase, catalase, and glutathione peroxidase. Accumulation of ROS can cause chemical and functional modification of DNA, RNA, protein, lipid and carbohydrate moieties, thereby resulting in cellular dysfunction. Our recent study proved that PPA could induce significant redox imbalance through GSH depletion, glutathione peroxidase inactivation, and potentiation of lipid peroxidation as a marker of oxidative stress [7].

Table 1 shows the potency of PPA in impairing the detoxification mechanisms in treated rats with 41% lower glutathione-s-transferase activity, a key family of enzymes that detoxify pro-oxidative compounds by coupling them to the body’s main antioxidant, GSH. This could support, at least in part, that redox imbalance may cause the neuronal insult and dysfunction seen in autistic patients and animal models. These suggestions are consistent with the study of James *et al.*[26], in which differences in allele frequency and/or significant gene interaction between individuals with autism and typically developing control subjects were found with genes encoding GSH-s-transferase. In the present study, NAC significantly counteracted PPA-induced oxidative stress. Although, GSH-s-transferase activities in NAC (protected and therapeutic) groups are lower compared to control, both show much higher activities compared to PPA-intoxicated group.

**Table 1:** One-way ANOVA test between serotonin (ng/100 mg), IFN-γ (pg/100 mg) and GST (μmol/min/100 mg) in control, PPA-intoxicated and NAC protected and treated rats

**Parameters**	**Groups**	**Min**	**Max**	**Mean ± SD**	***P *value**
Serotonin (ng/100 mg)	Control	3.41	4.80	4.12 ± 0.51	0.001
	PPA	2.32	3.03	2.76 ± 0.29 ^a^	
	NAC protective	3.12	3.94	3.53 ± 0.27 ^a^	
	NAC therapeutic	2.58	3.25	2.94 ± 0.27 ^a^	
IFN-γ (pg/100 mg)	Control	88.27	115.64	99.91 ± 8.84	0.001
	PPA	177.34	194.59	184.72 ± 6.24 ^a^	
	NAC protective	149.07	163.59	156.99 ± 5.23 ^a^	
	NAC therapeutic	160.57	171.66	167.60 ± 4.01 ^a^	
GST (μmol/min/100 mg)	Control	0.78	0.95	0.85 ± 0.06	0.001
	PPA	0.44	0.55	0.50 ± 0.04 ^a^	
	NAC protective	0.62	0.77	0.69 ± 0.05 ^a^	
	NAC therapeutic	0.59	0.70	0.65 ± 0.04 ^a^	

Significant levels between the three groups are illustrated as superscript letters when *P* <0.05.

**Figure 1:**
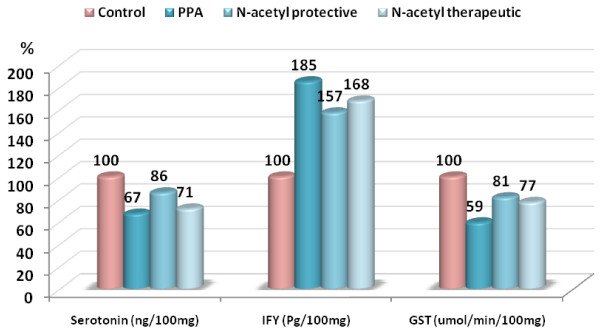
Percentage change of serotonin, IFN-γ and GST in PPA-intoxicated, NAC protected and treated groups, compared to control.

The anti-inflammatory effect of NAC reported in the present study, demonstrated in Table 1 and Figure 1 as significant reduction in the levels of IFN-γ, could be supported through considering the previous reports about the rationale use of NAC in Parkinson’s disease patients which was based on the capacity of this compound to reduce oxidative stress and inflammation. In fact, NAC reduces cellular production of proinflammatory mediators as IL-6 and stimulates the cellular GSH system [27]. Another anti-inflammatory effect of NAC was proved to be through the inhibition of cyclooxygenase products like prostaglandins [28] and down-regulation of TNF-α production and their soluble receptors, as well as TGF-B [29]. The protective effect of NAC reported in the present study is in good agreement with the work of Lante *et al.*[30], that pre-treatment with NAC prevented oxidative stress and loss of long-term potentiation following exposure to prenatal inflammation. Furthermore, lipopolysaccharide treatment results in inhibited oligodendroglial cell development and myelination that is attenuated by NAC administration in rat mixed glial cultures [31].

ROS have been shown to modulate levels and activity of noradrenaline (norepinephrine), serotonin, dopamine and glutamate, the principal neurotransmitters involved in the neurobiology of many brain diseases. These observations introduce new potential targets for the development of therapeutic interventions based on antioxidant compounds such as NAC [32]. In addition to the effects on oxidative balance, alterations in cysteine levels have also been shown to modulate neurotransmitter pathways, including glutamate and dopamine [33,34]. Cysteine assists in the regulation of neuronal intra- and extracellular exchange of glutamate through the cysteine-glutamate located on glial cells [35]. The dimer, cystine, is taken up by astrocytes and exchanged for glutamate, which is released into the extracellular space. This free glutamate appears to stimulate inhibitory metabotropic glutamate receptors on glutamatergic nerve terminals and thereby reduce the synaptic release of glutamate [36]. Given that relation, the amount of cysteine in the system as well as the feedback via GSH production by neurons may directly regulate the amount of glutamate present in the extracellular space. Furthermore, GSH itself has been shown to potentiate brain *N*-methyl-d-aspartate receptor response to glutamate in rats [37,38]. Changes in the levels of neuronal GSH may not only alter available glutamate levels, but also have direct consequences on glutamatergic function. NAC as a source for cysteine could be effective in ameliorating the impaired neurotransmission pathways induced by PPA neurotoxicity [7]. In the present study, NAC was effective in inducing serotonin levels, the 33% decrease of serotonin recorded in PPA-treated rats was only reduced to a value of 14% and 29% decrease in NAC-protected and treated rats, respectively. The recorded protective effect of NAC reported in the present study could be supported through the work of Lafleur *et al.*[39] in which NAC augmentation of serotonin receptor inhibitor treatment resulted in decreased symptoms in a single obsessive-compulsive disorder case.

**Figure 2:**
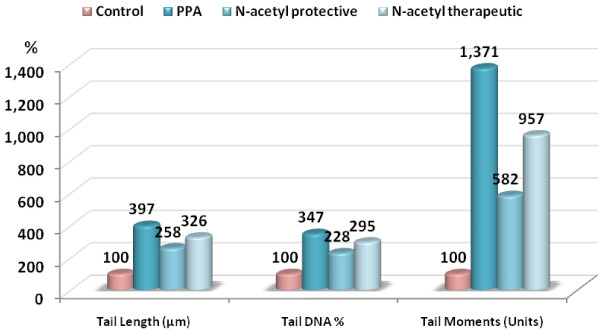
Percentage change of tail length, tail DNA% and tail moment in PPA-intoxicated, NAC-protected and treated groups compared to control.

**Figure 3:**
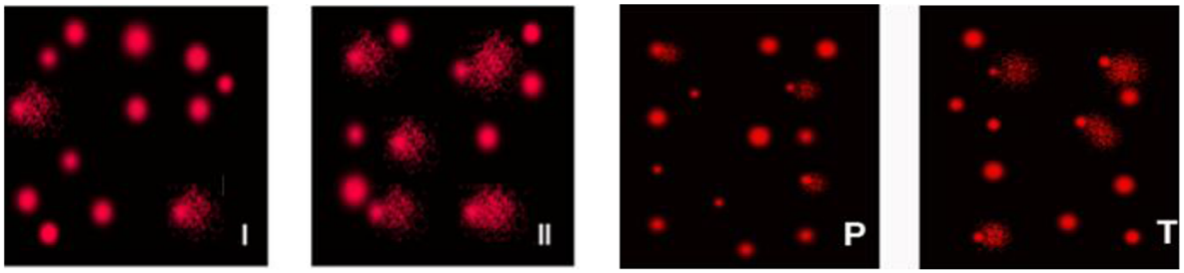
Comet DNA; I: Control group, II: Propionic acid treated group, P: NAC protective, and T: NAC therapeutic.

Table 2 and Figures 2 and 3 show the protective and therapeutic effects of NAC against PPA neurotoxicity which was demonstrated as a significant increase in the tail length (297%), tail DNA % (247%), and a dramatic increase in the tail moment (1271%). NAC given before PPA was effective in reducing the increase in these three parameters to values of 158%, 128% and 482%, respectively. While NAC gave almost 60% reduction in tail moment as marker of DNA double strand breaks, it induced only 30% therapeutic potency against PPA-induced effect. The recorded effect of NAC to protect against PPA neurotoxicity could find a support in the work of Reliene *et al.*[40] in which they recorded a protective effect of NAC against ionizing radiation-induced DNA damage. Similar to our obtained findings, exposure of human liver carcinoma (HepG2) cells to 30 mg/mL of PbNO_3_ caused a substantial level of cell death associated with a high degree of DNA damage, manifested by an increase in percentage of DNA in the tail and olive tail moment. Interestingly, co-treatment with a physiologic dose (500 mM) of NAC markedly lowered the cytogenotoxic effects of PbNO_3_*in vitro*. Other studies indicated that NAC protects macrophage cell line (THP-1) against diesel exhaust particle chemicals [17]. Yang and his co-workers [41] also reported that NAC lowers DNA damage produced by water-soluble cigarette smoke in human lymphoid cells containing Epstein-Barr virus episomes. *In vitro* studies suggest that the toxicity and the increase in lipid peroxidation induced by lead in cancer cell lines can be ameliorated by antioxidants such as NAC [42].

**Table 2:** One-way ANOVA test between tail length (μm), tail DNA % and tail moments (units) in control, PPA-intoxicated and NAC-protected and treated rats

**Parameters**	**Groups**	**Min**	**Max**	**Mean ± SD**	***P *value**
Tail length (μm)	Control	102.18	253.79	1.25 ± 0.16	0.001
	PPA	112.06	372.45	4.96 ± 0.28 ^a^	
	NAC protective	102.18	288.41	3.22 ± 0.23 ^a^	
	NAC therapeutic	29.66	125.25	4.07 ± 0.14 ^a^	
Tail DNA %	Control	48.99	95.00	1.40 ± 0.17	0.001
	PPA	22.00	62.99	4.85 ± 0.27 ^a^	
	NAC protective	18.20	58.80	3.18 ± 0.08 ^a^	
	NAC therapeutic	9.70	26.60	4.12 ± 0.15 ^a^	
Tail moments (units)	Control	34.48	37.06	1.76 ± 0.37	0.001
	PPA	23.27	31.03	24.07 ± 1.87 ^a^	
	NAC protective	32.32	36.20	10.23 ± 0.47 ^a^	
	NAC therapeutic	20.68	28.01	16.80 ± 1.16 ^a^	

Significant levels between the three groups are illustrated as superscript letters when *P* <0.05.

**Figure 4:**
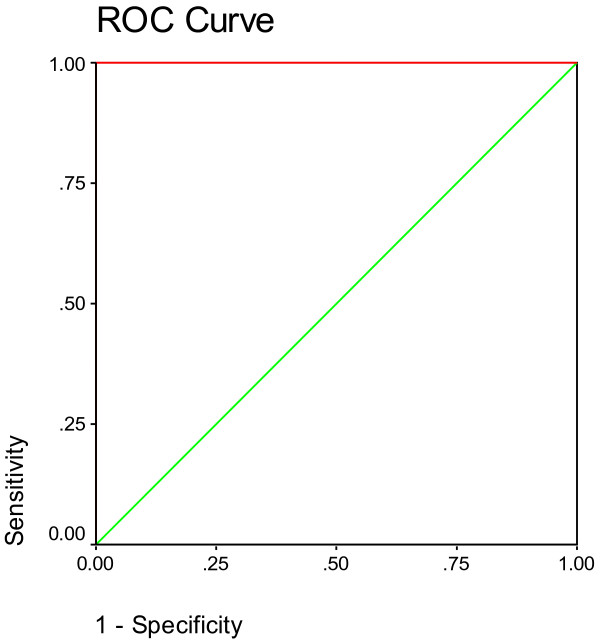
ROC curve of serotonin (ng/100 mg), IFN-γ (pg/100 mg) and GST (μmol/min/100 mg) in PPA and NAC therapeutic groups.

**Figure 5:**
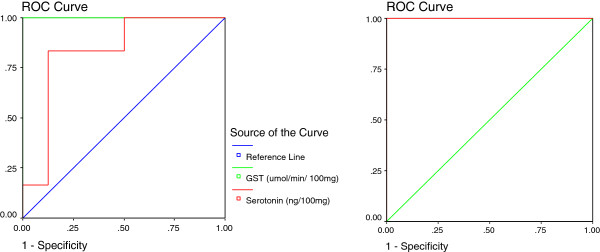
ROC curve of serotonin (ng/100 mg), IFN-γ (pg/100 mg) and GST (μmol/min/100 mg) in NAC protective group.

**Figure 6:**
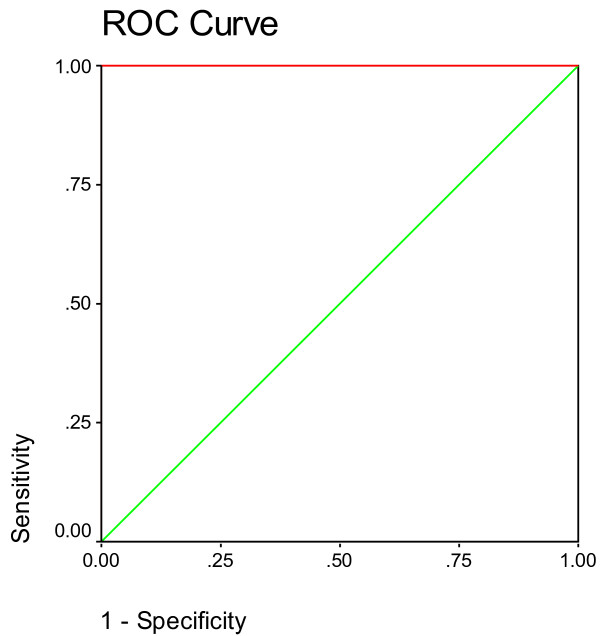
ROC curve of tail length (μm), tail DNA % and tail moments (units) in PPA, NAC protective and NAC therapeutic groups.

Figures 4, 5 and 6 demonstrate the ROC analysis of the measured parameters in PPA-intoxicated, NAC-protected and NAC-therapeutically treated rats. It could be easily noted that all the measured parameters show satisfactory values of sensitivity and specificity. This could ascertain the role of the measured parameters in testing the neurotoxic effect of PPA and the effectiveness of NAC in ameliorating the toxic effect of this short chain fatty acid.

**Figure 7:**
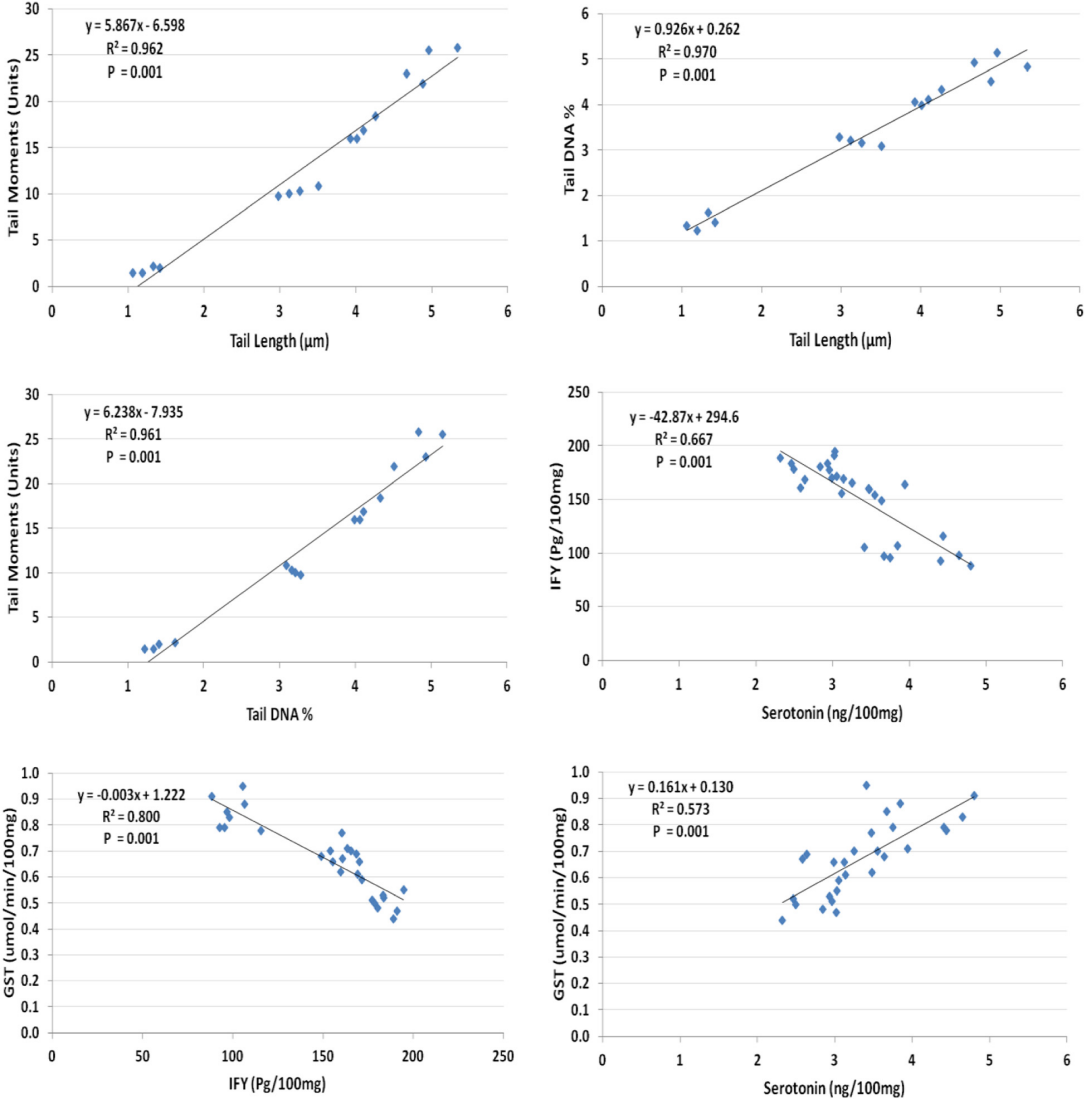
Pearson’s positive and negative correlations between the measured parameters.

The Pearson’s positive correlation seen in Figure 7 between serotonin and glutathione-s-transferase and the negative correlation between IFN-γ and glutathione-s-transferase could prove that the impairment of the detoxification mechanism represented by lower glutathione-s-transferase activity with PPA is related to alteration of brain neurochemistry represented by serotonin and induction of inflammatory responses represented by IFN-γ.

Taking into account the low dose and the short duration of NAC treatment tested in the present study, NAC supplementation could be suggested as a preventive strategy in case of PPA neurotoxicity as an etiological factor recently related to autism [7,43]. The effectiveness of NAC has also been linked to its antioxidative properties. NAC increases cysteine levels, thereby increasing the size of the glutathione pool, compared to other antioxidants examined in autism including omega-3 fatty acid and vitamin C which only promote GSH recycling by facilitating the conversion of GSSG into GSH without changing the size of the GSH pool. The suggested use of NAC could find a support in the recent randomized pilot investigation which proves the potential usefulness of NAC for treating irritability and behavioural disturbance in children with autism [12]. Hence, NAC treatment may be a promising therapeutic candidate for chemoprevention against PPA toxicity.

## Abbreviations

GSH: Glutathione; IFN-γ: Interferon-γ; IL-6: Interleukin-6; NAC: N-acetyl-cysteine; PPA: Propionic acid; ROS: Reactive oxygen species; TNF-α: Tumour necrosis factor-α

## Competing interests

The author(s) declare that they have no competing interests.

## Authors’ contributions

AA: Suggested the topic and co-drafted the manuscript, RB: Performed the practical work, AE: Design the work, drafted the manuscript and performed the statistical analysis. All authors read and approved the final manuscript.
